# Orthobiologics in Trauma: Current Trends, Future Directions, and Regenerative Strategies

**DOI:** 10.7759/cureus.108535

**Published:** 2026-05-09

**Authors:** Mohammed Al-Rumaih, Waleed Al-Hoshan, Mansour Al-Shehri, Salman Al-Harbi, Abdulrahman Al-Otaibi

**Affiliations:** 1 Department of Orthopaedic Surgery, Security Forces Hospital Program, Riyadh, SAU

**Keywords:** bone marrow aspirate concentrate (bmac), bone morphogenetic proteins, orthobiologics, platelet-rich plasma (prp), regenerative medicine, trauma

## Abstract

There is a paradigm shift in the field of regenerative medicine in trauma toward the application of orthobiologics, including platelet-rich plasma (PRP), bone marrow aspirate concentrate (BMAC), mesenchymal stem cells (MSCs), and bone morphogenetic proteins (BMPs). This review critically examines the clinical evidence, mechanisms of action, and potential of orthobiologics in the treatment of delayed unions, non-unions, acute fractures, and soft-tissue injuries. The literature indicates that BMPs are effective in bone repair, particularly in open fractures. In non-unions, PRP and BMAC/MSCs have demonstrated encouraging outcomes in bone healing and decreased revision surgeries. Although the evidence is encouraging, the use of these treatments is limited by the differences in clinical trials, preparation procedures, and standardization. Over the next few years, the development of precision medicine will lead to clinical successes, with biomarker-based treatments and next-generation cell-free therapies, including exosomes, potentially complementing existing therapies. The review also highlights the need for rigorous randomized controlled trials, protocols and longitudinal research to integrate these promising therapies into trauma treatment algorithms.

## Introduction and background

Orthobiologics are biological agents used to mediate tissue repair and regeneration [1]. They are based on autologous (e.g., the patient's own blood, bone marrow, or fat) or allogeneic sources and may consist of platelet-rich plasma (PRP), bone marrow aspirate concentrates (BMAC), mesenchymal stem/stromal cells (MSCs), growth factors (e.g., bone morphogenetic proteins [BMPs]), bioactive scaffolds, extracellular matrix, and even more recent gene-based technologies [1, 2]. Orthobiologics aim to alter the tissue environment, support natural repair processes, and restore function, rather than relying on surgical interventions that predominantly focus on mechanical fixation and symptom relief [3].

In the context of trauma, orthobiologics work to overcome limitations in fracture repair by enhancing the four overlapping stages: inflammation, soft-callus formation, hard-callus formation, and remodeling [4, 5]. Trauma to the bone and surrounding tissues affects local vascularity, progenitor cells, and cytokine activation, which can result in stalled advancement through these phases [4]. Orthobiologics help restore damaged tissue by providing scaffolds to direct cell migration and growth factors to promote osteoblast differentiation and neovascularization, as well as progenitor cells to downregulate inflammatory cytokines and drive endochondral ossification [6]. In acute fractures, open fractures, delayed unions, non-unions, post-traumatic cartilage and soft-tissue defects, they promote a biologic milieu in which conventional fixation alone may be inadequate, ultimately leading to more effective callus formation and repair [7]. Orthobiologics, therefore, provide a means to enhance the biological environment at the fracture site, potentially accelerating healing and improving clinical outcomes [3, 6].

Orthobiologics hold considerable promise for improving the healing of musculoskeletal tissues, including bone, cartilage, and soft tissues [6, 7], but very little clinical evidence supports their broad application. Many orthobiologic treatments are nevertheless currently being marketed in the orthopedic industry without strong clinical evidence supporting their effectiveness. This is further complicated by concerns about the safety, manufacturing consistency, and expected outcomes of these therapies. In addition, many orthobiologic therapies are still under investigation, and the long-term outcomes, risks, and complications associated with their use are poorly understood. This review will provide an overview of the evidence on the use of orthobiologics in musculoskeletal trauma. It will discuss the mechanisms of action, review the available evidence on safety and effectiveness, and outline the major knowledge gaps that must be addressed.

## Review

Materials and methods

We conducted a systematic literature review to identify peer-reviewed articles relevant to orthobiologics in trauma and musculoskeletal healing. The databases searched included PubMed/MEDLINE, Web of Science, and Google Scholar, starting January 2020 and through April 2026. We used a search strategy that incorporated controlled vocabulary (MeSH terms) and free-text keywords that mapped to orthobiologic therapies and trauma, refined with Boolean operators: ((“orthobiologic” OR “platelet-rich plasma” OR “PRP” OR mesenchymal stem cell OR MSC* OR bone morphogenetic protein OR BMAC OR bone marrow aspirate)). The reference lists of the relevant articles and grey literature were also manually screened to ensure all articles were covered.

We found 987 records in our initial search. After removing duplicates (n = 432), we screened 1155 articles by title and abstract for relevance to orthobiologic therapies in trauma. Of these, we selected 47 articles for full-text review based on their potential to address the use of orthobiologics in trauma.

Eligibility criteria included studies that were clinical trials, cohort studies, systematic reviews, or meta-analyses involving orthobiologic interventions for fracture healing, non-union, or trauma-related soft tissue repair in human subjects, with full-text availability. Based on these criteria, we included 17 studies in the final synthesis.

Results

Types of Orthobiologics and Mechanisms of Action

Orthobiologics cover a spectrum of biologically derived products designed to augment tissue healing and regeneration after trauma [3]. Major categories currently studied include:

Blood‑derived therapies (PRP): Platelet‑rich plasma (PRP) is an autologous blood derivative that concentrates platelets and their associated growth factors, including PDGF, TGF‑β, and VEGF, which are released upon activation to stimulate reparative processes such as chemotaxis, angiogenesis, and cell proliferation at injury sites [7]. These molecules initiate and amplify the early inflammatory phase of fracture healing by recruiting fibroblasts, promoting angiogenesis, and stimulating osteoblast differentiation, thereby accelerating soft-callus formation in preclinical models [8].

Recent studies have shown that PRP actively stimulates regenerative responses in fracture healing by activating specific signaling pathways that regenerate the extracellular matrix (ECM) and promote appropriate reparative cellular functions [7, 9]. The ECM provides a scaffolding structure for surrounding cells and a biochemical signaling center and is thus a key site of PRP bioactivity [10]. PRP triggers hyperstimulation of ECM synthesis, supplying the injury site with matrices rich in critical bioactive proteins and growth factors. Its anabolic effects thus augment the synthesis of vital structural constituents, such as proteoglycans, fibronectin, collagen, and other extracellular matrix proteins, which form a stable provisional scaffold necessary for tissue repair [7, 11].

Cell‑based therapies (MSCs/BMAC): Mesenchymal stem/stromal cells (MSCs) are multipotent progenitor cells that can differentiate into osteogenic, chondrogenic, and fibroblastic lineages; they secrete immunomodulatory cytokines, chemokines, and growth factors, which together promote tissue regeneration and eliminate inflammation, which is fundamental to healthy bone healing [12]. Some of the most widely researched cellular orthobiologics used in trauma and regeneration include MSCs derived from bone marrow, adipose tissue, and perinatal sources [12, 13]. MSCs secrete an abundant repertoire of cytokines, chemokines, and extracellular vesicles that regulate the immune response, stimulate resident progenitor cells, and enhance angiogenesis by recruiting and polarizing macrophages toward pro-healing phenotypes. This paracrine crosstalk is an essential process underlying MSC-based enhancements in musculoskeletal repair [14].

Bone marrow aspirate concentrate (BMAC) is an autologous orthobiologic prepared by centrifugation of bone marrow aspirate to concentrate nucleated cells, especially MSCs, as well as platelets, growth factors, and cytokines [15]. The MSCs in BMAC have both trophic and immunomodulatory effects, with a range of growth factors, including PDGF, TGF-β, VEGF and IGF, that promote local cell proliferation, angiogenesis and extracellular matrix synthesis, which are involved in bone and soft tissue regeneration [15, 16]. These mechanisms also provide a paracrine signaling microenvironment that attracts endogenous progenitor cells and regulates inflammatory reactions to support healing [16].

Growth factors & recombinant proteins: Bone morphogenetic proteins (BMPs), including BMP-2 and BMP-7, are osteoinductive growth factors that bind BMP receptors on progenitor cells and activate SMAD signaling pathways, which induce osteoblast differentiation and matrix formation - essential processes in reparative bone formation [17]. Another important factor that affects the proliferation of endothelial cells and the formation of new blood vessels is Vascular Endothelial Growth Factor (VEGF). This growth factor plays a crucial role in stimulating the growth and division of endothelial cells, as well as in the process of neovascularization. By promoting these processes, VEGF ensures that tissues receive an adequate supply of oxygen and nutrients, which are vital for effective tissue repair and regeneration. Growth factor signaling is carefully regulated in vivo, and exogenous expression often requires controlled delivery systems to prevent desensitization or fibrosis [18].

The conversion of growth factor-beta (TGF-β) family members, namely, TGF-β1 and TGF-β2, plays a critical role in bone remodeling and healing [19]. TGF-β1 is stored in platelets and bone cells and is released during bone resorption to mediate coupled bone formation [20]. It also regulates wound healing in soft tissues, though overexpression may enhance scar tissue formation and fibrosis [20]. TGF-β2 is found in cartilage and bone tissue, has a strong chondrogenic effect, and supports cartilage ECM deposition, making it potentially useful in the treatment of cartilage injury and osteoarthritis (Table 1) [21].

**Table 1:** Summary of Growth Factor-Based and Tissue-Derived Orthobiologics in Trauma BMP: Bone morphogenetic protein; VEGF: Vascular endothelial growth factor; MSC: Mesenchymal stem cell; TGF‑β: Transforming growth factor-β

Orthobiologic Agent	Source	Key Functional Components / Growth Factors	Primary Target Tissue(s)	Trauma / Regenerative Application
BMP‑2 BMP‑7	Recombinant proteins (rhBMPs)	BMP‑2, BMP‑7 (osteogenic growth factors)	Bone & osteoprogenitors	Nonunion, open fractures, bone reconstruction [17, 22]
VEGF	Platelets, MSCs, injury milieu	VEGF‑A	Endothelial cells & angiogenic zones	Critical in early fracture healing and large defect repair [18]
TGF‑β	Platelets, bone matrix	TGF‑β1, TGF‑β2, TGF‑β3	Cartilage, bone, connective tissues	Supportive in fracture callus and cartilage repair [20, 21]
Fibroblast Growth Factors (FGFs)	Platelets, mesenchymal cells	FGF‑2, FGF‑18 (various isoforms)	Mesenchymal progenitors, osteoblasts, chondrocytes	Early healing phases of bone/soft tissue trauma [23]

Biomaterials & scaffolds: Scaffold-based orthobiologics combine hydrogels, collagen matrices, and 3D-printed constructs to provide structural support and controlled delivery of the above-described biologics [24]. They are structural scaffolds that aid cellular adhesion, endogenous progenitor recruitment, and sustained local delivery of orthobiologic factors, effectively mediating between biological and mechanical repair demands [24]. Mechanically, these scaffolds provide osteoconductive surfaces and controlled delivery platforms to enhance cell and growth factor retention and bioactivity at the site of trauma, thereby supporting more effective tissue regeneration [25]. Injectable hydrogels loaded with BMPs, MSCs or growth factors can be implanted with minimal invasiveness and degrade at the same rate as healing pathways, leading to therapeutic concentrations of active molecules in the local environment and reducing the risk of ectopic bone formation [26].

Current Evidence: Applications of Orthobiologics in Trauma Healing

Recent evidence on the use of orthobiologics in trauma includes the following:

Acute fracture healing (closed and open fractures): In acute fractures, especially in long bones, the biological environment following injury is marked by inflammatory cues, progenitor cell recruitment, and rapid vascular ingrowth to facilitate callus formation, and orthobiologics seek to stimulate these events [1, 7]. PRP demonstrates inconsistent results in acute fracture healing. A study by Boonyanuwat et al. (2025) found that PRP shortens healing time in some populations, including patients with tibial shaft fractures, and improves mid-stage callus development [27]. According to Kale et al. (2024), PRP used as an adjunct reduces fracture-healing time and enhances bone mineral density in specific groups, such as those with tibial shaft fractures [8]. Systematic reviews confirm greater mid-stage callus development in less-energetic fractures, but the total union rates of closed fractures do not demonstrate uniform improvements [28].

BMPs still show the strongest data among orthobiologics in acute open fractures. BMP-2 efficacy in open tibial shaft fractures has been demonstrated in historical trials, and recent network meta-analyses confirm shorter healing times compared with usual care [29]. Another meta-analysis by Sakong et al. (2026) confirms that BMP supplementation reduces the healing period compared with standard care alone and is particularly beneficial in high-energy open injuries treated with intramedullary nailing [30].

BMAC and MSCs provide greater adjunctive support in the management of open fractures. Percutaneous injection of autologous bone marrow concentrate in the early treatment of Gustilo-Anderson Type II or III open tibial fractures results in high rates of union and a markedly lower rate of infection and delayed union compared with historical controls [30,31]. This early grafting approach directly targets local depletion of progenitor cells and impaired vascularity at the fracture site [32].

Delayed union and non-union: Delayed union and non-union are clinical conditions in which the normal progression of inflammation, callus formation, and remodeling halts, and these conditions are among the strongest predictors of orthobiologic augmentation [33]. Non-union, by definition, is a complication of failure to heal within a biologically reasonable period, which may require the use of biological stimulation along with mechanical stability, and PRP, BMPs, and BMAC have been examined in this context [28].

PRP, when used alone or in combination with bone grafting, has shown promise in enhancing healing in delayed union and nonunion, with higher radiographic union rates and shorter time to consolidation reported in several case series. A meta‑analysis by Li et al. (2022) found that PRP injected at delayed union or non-union sites led to a healing rate of around 85.80% compared to control groups near 60.76%, and PRP groups often reported improved pain scores [34]. Niu et al. (2025) also reported that PRP‑augmented iliac bone grafts in atrophic tibial non-unions showed shorter healing times and improved functional outcomes compared to historical controls treated without PRP [35]. A network meta-analysis of 15 randomized controlled trials involving 1,286 patients demonstrated that PRP and BMP are superior to standard care for both healing time and union rates [29]. Moreover, MSC therapy yields an eventual healing rate of 91% and reduces mean union time by approximately 0.54 months with low complication rates according to a meta-analysis by Cui et al. (2025) [36].

Tendon & ligament injury: Another significant area of orthobiologics research in the context of trauma care is soft-tissue injuries, including tendon and ligament tears. The most commonly researched therapy is PRP because it is easy to prepare, autologous, and contains growth factors. Preclinical evidence indicates that PRP affects tendon biology by releasing chemotactic factors that trigger tenocyte proliferation, collagen formation and angiogenesis, which are essential for early- and mid-phase tendon healing [7]. Clinical outcomes are variable, however. Clinical trials indicate some pain and functional improvements in chronic tendinopathies such as lateral epicondylitis or partial rotator cuff disease [37], although others have found only minimal differences compared with controls, suggesting heterogeneity in PRP preparations and procedures [38].

Complex trauma & segmental bone loss: Complex trauma, such as segmental bone loss, which is present in high-energy injuries, open fractures, and composite defects, is also problematic because the biological deficit is worsened by mechanical instability and loss of soft tissues, and mechanical and regenerative interventions may be necessary [39]. In such cases, orthobiologics are not administered alone. Liu et al. (2022) found that PRP-impregnated gelatin microspheres in composite scaffolds were better in survival, proliferation, migration, and osteogenic differentiation of MSCs than control scaffolds in a preclinical large bone defect model [40]. The second approach is to incorporate orthobiologics, such as PRP, into bioactive Glass, hydroxyapatite, or chitosan carriers to improve scaffold performance. According to Meglei et al. (2025), PRP with osteoconductive carriers was associated with much higher rates of defect closure and new bone mineralization than a scaffold-only model [41]. Long-bone defect models of PRP-impregnated scaffolds loaded with human MSCs demonstrated considerable cellular growth and tissue regeneration at 12 weeks, highlighting the promise of engineered constructs in clinically demanding situations (Table 2) [42].

**Table 2:** Clinical Evidence of Orthobiologics in Trauma Sub‑Domains PRP: Platelet-rich plasma; BMP: Bone morphogenetic protein; BMAC: Bone marrow aspirate concentrate; MSC: Mesenchymal stem cell

Orthobiologic	Sub‑types	Evidence Level	Clinical Outcomes	References
PRP	Leukocyte‑rich PRP Leukocyte‑poor PRP	RCTs, Cohort Studies	Mixed results in acute fractures and non‑unions due to protocol variability. Some studies show improvement in radiographic healing time	[1, 27, 28]
		RCT	PRP reduces healing time and improves mid-stage callus formation in tibial shaft fractures	[27]
		Comprehensive review	PRP improves bone mineral density and shortens fracture healing duration in tibial fractures	[8]
BMPs	BMP‑2	Network Meta-analysis	BMP supplementation shortens healing time in open fractures, particularly in high-energy injuries treated with intramedullary nailing.	[30]
BMAC	BMAC from iliac crest BMAC from adipose tissue	Systematic review	High success in nonunions, with improved healing and consolidation. Effective in critical size defects when combined with scaffolds.	[28]
		Case Series	Early grafting with BMAC achieves high union rates and reduces infection and delayed union in open tibial fractures.	[31]
PRP + Bone Graft	PRP + autologous graft PRP + synthetic graft	Meta-analysis, Cohort Studies	Accelerated healing in delayed union fractures; enhanced bone consolidation when combined with autologous bone graft.	[34, 35]
MSCs	BMAC Adipose-derived MSCs	Systematic review and meta-analysis	High union rates; reduced delayed union	[36]
Composite Scaffolds + Orthobiologics	PRP‑laden Gelatin Microspheres PRP + Bioactive Glass	Preclinical Data	Improved MSC survival, proliferation, and osteogenic differentiation in large bone defect models; higher osteogenic differentiation and tissue recovery.	[40]
PRP + Osteoconductive Carriers	PRP + Hydroxyapatite, Chitosan	Preclinical Data	Enhanced defect closure and bone mineralization in segmental bone defects; increased tissue recovery over 12 weeks in scaffold‑MSCs studies.	[41]

Limitations and Challenges

Orthobiologics in trauma have several significant limitations that impede widespread standardization and optimal clinical use, despite their potential. Heterogeneity in product preparation, dosing, nomenclature and reporting of outcomes is the greatest challenge. PRP preparations, e.g., are highly heterogeneous in platelet-based, leukocyte-based, and centrifugation procedures, and may exhibit growth-factor release patterns that vary by up to 40% across the literature [43]. Equally, BMAC and MSC products differ because the techniques for extracting donor sites, processing time, and end-cell viability are seldom standardized, and comparisons across trials are complicated [44].

Robust clinical evidence has lagged behind the popularity of orthobiologics. This is because unregulated clinics may provide cell therapies and biologic injections without substantial supporting evidence, resulting in mixed patient outcomes and potentially unsafe practices [1, 45]. Further, the regulatory pathways are complex: an FDA/HCT/P regulation may classify a product as minimally manipulated or more than minimally manipulated, and this distinction can have a significant impact on approval, clinical labeling, and surveillance [45, 46].

Future Directions and Regenerative Strategies

The orthobiologics field is moving towards precision and personalized medicine, where the biological signatures of patients, including inflammatory markers, senescence markers, and genetic predispositions, are matched to regenerative therapies rather than a one-size-fits-all approach. It is further shown that combining molecular biomarkers, high-resolution imaging, and artificial intelligence can inform rational dosing and optimize therapeutic combinations to enhance prognostication and predictive clinical response in musculoskeletal regenerative therapies [26].

A highly intriguing field of study is the production of cell-free therapies, specifically exosomes and extracellular vesicles (EVs), which are natural signaling molecules in cell-to-cell communication and can carry bioactive cargo that induces inflammation, promotes angiogenesis, and elicits osteogenic and chondrogenic responses [47]. Preclinical studies show that EV-based therapies can suppress inflammation, promote cartilage and bone regeneration, regulate matrix metabolism, and modulate tissue-specific regenerative pathways, potentially with reduced safety risks compared with whole-cell therapies [47, 48].

## Conclusions

Orthobiologics, including PRP, BMAC/MSCs, and BMPs, are changing the paradigm of trauma treatment toward the regenerative approach, particularly in delayed and nonunion fractures, where the agents improve union rates and reduce the healing period. They also have potential use as adjuncts to acute fractures and soft-tissue defects, complementing biological healing in situations where conventional treatments are likely to be insufficient. Orthobiologics are not widely used in the management of acute fractures, yet they help minimize the risk of revision surgery and enhance recovery in complicated cases. The future of orthobiologics is linked to standardized protocols, personalized medicine, and the creation of sophisticated regenerative platforms, including exosomes and engineered scaffolds.
